# CircRNA-Mediated Regulation of Angiogenesis: A New Chapter in Cancer Biology

**DOI:** 10.3389/fonc.2021.553706

**Published:** 2021-03-10

**Authors:** Shaotao Jiang, Rongdang Fu, Jiewei Shi, Huijie Wu, Jialuo Mai, Xuefeng Hua, Huan Chen, Jie Liu, Minqiang Lu, Ning Li

**Affiliations:** ^1^ Department of HBP SURGERY II, Guangzhou First People’s Hospital, School of Medicine, South China University of Technology, Guangzhou, China; ^2^ Department of Hepatic Surgery, The First People’s Hospital of Foshan, Affiliated Foshan Hospital of Sun Yat-sen University, Foshan, China; ^3^ Department of General Surgery, Guangzhou First People’s Hospital, Guangzhou Medical University, Guangzhou, China; ^4^ Department of Obstetrics, The First People’s Hospital of Foshan, Affiliated Foshan Hospital of Sun Yat-sen University, Foshan, China

**Keywords:** circRNA, miRNA, tumor angiogenesis, VEGF, signaling pathways

## Abstract

Angiogenesis is necessary for carcinoma progression and is regulated by a variety of pro- and anti-angiogenesis factors. CircRNAs are RNA molecules that do not have a 5’-cap or a 3’-polyA tail and are involved in a variety of biological functions. While circRNA-mediated regulation of tumor angiogenesis has received much attention, the detailed biological regulatory mechanism remains unclear. In this review, we investigated circRNAs in tumor angiogenesis from multiple perspectives, including its upstream and downstream factors. We believe that circRNAs have natural advantages and great potential for the diagnosis and treatment of tumors, which deserves further exploration.

## Introduction

Angiogenesis is characterized by the proliferation, differentiation and migration of endothelial cells (ECs) on the basis of existing capillaries or venules to generate new blood vessels (1–3). In normal circumstances, blood vessels are regulated by multiple angiogenic factors that promote or inhibit angiogenesis to maintain homeostasis. However, active proliferation and increased energy metabolism are characteristics of a tumor. Primary or metastatic cancer relies on the angiogenesis and formation of a rich network of blood vessels. In response to its own cell necrosis, tumor cells regulate the microenvironment by releasing pro-factors or by blocking the release of anti-angiogenic factors. By activating the “angiogenesis switch” in the tumor, the vascular system is stimulated to sprout new blood vessels (3, 4), so as to obtain more energy and oxygen (5), and to promote the proliferation of the tumor. Meanwhile, tumor cells spread and metastasize in other parts of the body. Therefore, inhibiting angiogenesis has become an important target for cancer therapy and has stimulated the drive to explain the mechanism of tumor angiogenesis.

A number of pro-angiogenic factors have been identified, including VEGF (6, 7), angiopoietin (8), matrix metalloproteinases (MMPs) (9, 10), and fibroblast growth factors (FGF) (11). VEGF specifically promotes vascular endothelial growth by promoting mitosis. Angiopoietins (8) are growth factors secreted by vascular endothelium that regulate vascular maturation and remodeling. Two important angiopoietins are Ang-1 and Ang-2 (12, 13). The balance of Ang-1 and Ang-2 in endothelial cells is key to normal angiogenesis. FGF (11) is a low molecular weight polypeptide growth factor with a specific structure, most of which can bind to heparin. FGF contains secreted signaling peptides that are secreted into the extracellular matrix (ECM) and can bind to acetylheparin aminoglycan. MMPs are proteases that hydrolyze ECM and remodel angiogenic basement membrane (14).

In contrast to the pro-angiogenic factors, endostatin, arrestin, and angiostatin have been shown to be antiangiogenic factors. Endostatin, a peptide of collagen XVIII, contains a zinc-binding domain and an arginine-binding domain, which allows it to bind to heparin and is important for heparin’s antiangiogenic activity (15). Arrestin selectively inhibits endothelial cell proliferation and migration and thus represses angiogenesis (16). Angiostatin (17) is the product of fibrinogen lyolysis. Interestingly, fibrinogen itself has no inhibitory effect on angiogenesis but can directly bind to ATP synthase to trigger apoptosis of endothelial cells, possibly by lowering the pH value in cells (18).

Circular RNAs (CircRNAs) are a type of long non-coding RNA (lncRNA) first identified in plant studies and thought to be a class of viroids (19). Because of low expression in cells, circRNAs were initially thought to be an error in RNA splicing. Next-generation sequencing techniques that do not rely on the 3’-polyA tail have been used to find extensive circRNAs in eukaryotic cells. With the development of deep sequencing, more and more RNA transcripts have been discovered, and the nonstandard pattern of RNA splicing leads to multiple subtypes of circRNAs (20). As the number of identified circRNAs increased, more of them were found to be biologically stable, and many circRNA transcripts were more abundant than their associated mRNA transcripts (21). Hansen and Memczak et al. first demonstrated a function of circRNAs, showing that circRNAs act as a sponge for miR-7 (22, 23). Since then, circRNAs have received extensive attention, and their characteristics and potential applications in clinical diagnosis and treatment have been explored.

It has been previously shown that circRNAs play an important role in tumor growth, angiogenesis, metastasis, recurrence, and antitumor therapy (24). CircRNAs regulate VEGFR-related pathways through adsorption of miRNA to affect tumor angiogenesis. CircRNAs have also been shown to be involved in regulating the tumor microenvironment (25). CircRNAs are highly abundant and stable, conserved in evolutionary species, and widely present in various body fluids. The exploration of tumor angiogenesis-related circRNAs as biomarkers or targets will open new possibilities for anti-tumor treatment strategies. Here, we focus on the biomolecular mechanisms of circRNA in tumor angiogenesis.

## CircRNA

### Biogenesis and Characteristics of circRNAs

After the removal of introns by enzyme-catalyzed precursor mRNA, selective splicing of exons in turn to form mature mRNA is common. Unlike typical mRNA splicing, circRNAs are produced by a back-splicing process, in which the downstream 5’ splicing site and the upstream 3’ splicing site are connected to form a single-chain covalently closed ring. The spliceosome then removes all or part of the introns and joins the remaining sequences. Three kinds of circRNAs are then produced, including exonic circRNA, intronic circRNA, and exon-intron circRNA (20, 26, 27).

The mechanism of circRNA formation is one of the basic scientific questions underpinning the study of circRNAs. Zhang et al. showed that the formation of circRNAs was determined by rapid transcription, the reverse complementary sequence in RNA, and the effect of long-term accumulation in cells (28). circRNA predictive analysis combined with the technique of long-fragment sequencing revealed that there were many selective splicing modes of circRNA (29). In addition, the formation of circRNAs is closely related to their selective splicing patterns and cell types (30). Besides, RNA binding proteins are involved in the formation of circRNAs. In drosophila, the splicing factor Muscleblind (Mbl) promotes the formation of circMbl from its own precursor mRNA (31). In general, the formation of circRNAs is parallel to the linear RNA transcription, which is related to the transcription speed of corresponding genes. Reverse complementary sequences or RBP binding sequences are important prerequisites for the formation of circRNAs. One gene may correspond to a variety of molecular forms of circRNAs.

### Biological Functions of circRNAs

CircRNAs play an important role in tumor growth, angiogenesis, metastasis, recurrence, and antitumor therapy through multiple functions (24). Recent studies suggest that circRNAs act as sponges to bind and block miRNAs, or as competing endogenous RNA (ceRNA) molecules (22, 23, 32). Previously, miRNAs have been shown to bind directly to their target mRNA in the form of base pairing, leading to cleavage of the mRNA transcript or inhibition of mRNA translation (33). Furthermore, RNA binding proteins regulate disease progression by directly targeting circRNAs (34–36). Additionally, circRNAs can compete with linear transcripts for splicing sites during reverse back-splicing (31). On the one hand, when more exons form circRNAs, the mRNA is reduced; on the other hand, circRNAs containing introns directly bind the U1 component in the spliceosome to recruit RNA polymerase II, thereby upregulating expression of the target gene (28). Interestingly, circRNAs have long been considered a non-coding RNA, but that has changed. There is an m6A modification for circRNAs that promotes translation (37). Additionally, the circRNA, circ-FBXW7, directly encodes the protein FBXW7-185aa and cooperates with the FBXW7 protein in linear transcripts to stabilize c-Myc and inhibit the occurrence and progression of malignant glioma (38). Furthermore, Pamudurti et al. found that a large amount of circRNA translated proteins or peptides were found in the *Drosophila* brain (39).

## The Interaction Between miRNAs and circRNAs in Tumor Angiogenesis

The primary mechanisms by which circRNAs regulate tumor angiogenesis is by functioning as a targeted sponge for miRNAs, by binding and blocking miRNAs, or by acting as competing endogenous RNA molecules (22, 23, 32). The regulation of miRNAs in tumor angiogenesis has been previously characterized (40). miRNAs directly target the 3’UTR region of the transcripts of pro-angiogenic or anti-angiogenic factors, resulting in the inhibition of mRNA translation and degradation of the mRNA (33). It has been shown that miRNA transcription is downregulated in tumor-related ECs. The reduction of competing miRNAs, which is a target of the 3’UTR region of VEGF-A mRNA, upregulate VEGF-A expression and promotes angiogenesis through VEGF/VEGFR-2 signaling pathways (41). In this study, we reviewed the circRNAs associated with angiogenesis and summarized their expression patterns, mechanism, and functions in tumor cells in **Table 1**.

**Table 1:** The expression patterns, mechanism, and functions of circRNAs associated with tumor angiogenesis.

CircRNA	Expression	Mechanism	Function	Origin	Ref
circRNA-MYLK	up	miR-29a/VEGFA/VEGFR2/Ras/ERK signaling pathway	proliferation, migration, tube formation of HUVEC and rearranged cytoskeleton	Bladder Cancer	(42)
CircHIPK3 /BCRC-2 /hsa_circ_0000284	down	miR-558/HPSE	migration, invasion, and angiogenesis	bladder cancer	(43)
Circ-ASH2L	up	miR-34a/Notch1	invasion, proliferation and angiogenesis	Pancreatic Ductal Adenocarcinoma	(44)
hsa_circRNA_002178 /hsa_circ_0000519	up	miR-328-3p/COL1A1	cell viability, energy metabolism and tube formation ability	breast cancer	(45)
CircSMARCA5 /hsa_circ_0001445	down	Splicing Factors SRSF1/VEGFA	cells migration and angiogenesis	glioblastoma multiforme	(46, 47)
circ-SHKBP1 /hsa_circ_0000936	up	miR-544a/FOXP1/miR-379/FOXP2/AGGF1	viability, migration, and tube formation of GEC	GECs	(48)
circ_002136	up	FUS/circ_002136/miR-138-5p/SOX13/SPON2	viability, migration and tube formation	GECs	(49)
circ-DICER1	up	MOV10/circ-DICER1/miR-103a-3p/miR-382-5p/ZIC4/Hsp90β/PI3K/Akt	cell viability, migration, and tube formation of GECs	GECs	(50)
Exosome has_circRNA_100338	up	VE-Cadherin and ZO-1	cell proliferation, angiogenesis, permeability, and vasculogenic mimicry formation ability of HUVECs	HCC	(51)
hsa_circ_0003575	up	potential circRNA-miRNA-mRNA network	proliferation and angiogenesis ability of HUVECs	HUVECs	(52)
hsa_circ_0010729	up	miR-186/HIF-1α Axis	vascular endothelial cell proliferation and apoptosis	HUVECs	(53)
cZNF609	up	miR-615-5p/MEF2A	retinal vessel loss and suppressed pathological angiogenesis	high glucose and hypoxia stress	(54)
circNfix /hsa_circ_0005660	up	miR-214/Gsk3β/β-catenin/Meis1(TF) Ybx1,Nedd4l cyclin A2,cyclin B1	proliferation angiogenesis and apoptosis	adult heart in humans, rats, and mice	(55)
circHIPK3	up	miR-30a-3p/VEGF-C, FZD4, and WNT2	cell viability, proliferation, migration, and tube formation	diabetic retinas and retinal endothelial cells	(56)
hsa_circ_0074834	down	microRNA-942-5p/ZEB1/VEGF	promote osteogenic differentiation of BMSCs and the repair of bone defects	BMSC	(57)
Circ_0063517	down	miR-31-5p-ETBR	growth, migration, and angiogenesis	placenta tissue of PE	(58)

### Pro-angiogenic-Associated circRNAs

#### CircRNA-MYLK

**Figure 1:**
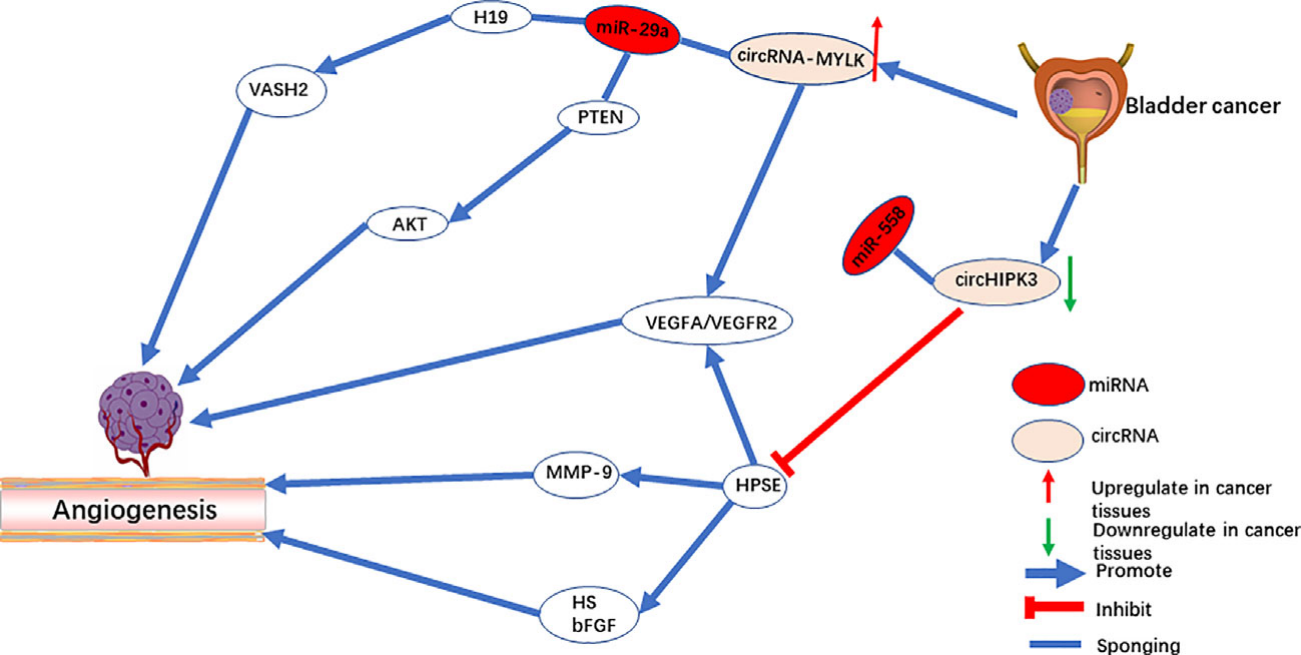
The role of circRNAs in pro-angiogenesis and anti-angiogenesis in bladder cancer.

CircRNA-MYLK is an oncogene in bladder cancer, and it activates the VEGF-A/VEGFR-2 signaling pathway by functioning as a sponge for miR-29a to up-regulate VEGFA expression (42). *In vitro*, overexpression of circRNA-MYLK promotes the ability of HUVECs to form blood vessels and rearrange the cytoskeleton. *In vivo*, upregulation of circRNA-MYLK promotes tumor progression and is a predictor of poor prognosis (**Figure 1**). In addition, studies have also shown that down-regulation of cell-derived microvesicle miR-29a relieves suppression of VEGFA and promotes angiogenesis in gastric cancer (59). The animal model demonstrates that angiogenesis can be inhibited by microvesicles rich with miR-29a. Peng et al. revealed that the lncRNA H19 acts as a miRNA sponge of miR-29a. Downregulating miR-29a promotes angiogenesis by targeting the 3’-UTR region of VASH2 (60). On the contrary, Wang et al. reported that miR-29a serves as an oncogene that activates the AKT pathway by targeting PTEN in endothelial cells and promoting tumor angiogenesis (61). Meanwhile, both miR-29a (62) and miR-362-3P (63) can be modulated by circRNA-MYLK, which up-regulates the expression of downstream Rab23 and promotes the progression of tumors. Therefore, whether circRNA-MYLK promotes tumor angiogenesis remains an open question, and further investigation is required to identify its mechanism in tumors in other than bladder cancer.

#### Circ-ASH2L

**Figure 2:**
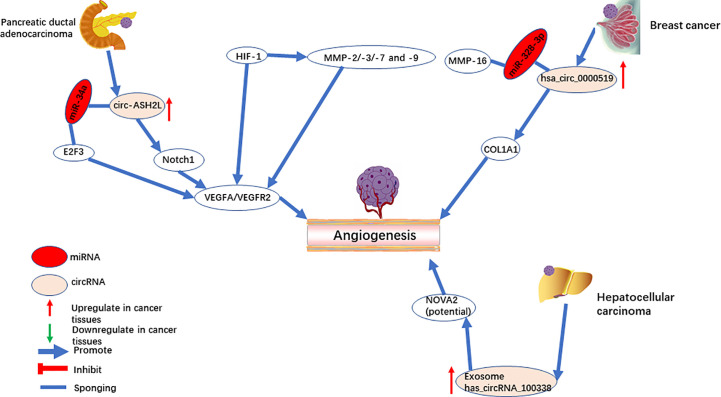
CircRNAs mediate angiogenesis in pancreatic ductal adenocarcinoma, breast cancer, and hepatocellular carcinoma.

Circ-ASH2L was first identified for promoting tumor angiogenesis in pancreatic ductal adenocarcinoma (44). As an oncogene, it was found in the RIP experiment to be a sponge for miR-34a. miR-34a has been widely reported to inhibit angiogenesis by repressing the Notch1 signaling pathway (64, 65). In the study of Chen et al. (44), circ-ASH2L also promoted angiogenesis by activating the Notch1 signaling pathway. *In vitro* experiments verified that circ-ASH2L sequesters miR-34a to increase the downstream expression of VEGF through the Notch1 signaling pathway (**Figure 2**). Meanwhile, miR-34a down-regulates VEGF expression through another axis that inhibits the translation and degradation of the E2F3 mRNA in head and neck squamous cell carcinoma (66).

#### Hsa_circRNA_002178 (hsa_circ_0000519)

Hsa_circ_0000519, a circular transcript of RPPH1, is located on chromosome chr14:20811436-20811534. Hsa_circ_0000519 was upregulated in breast cancer and was suggested as a marker of poor prognosis (45). Knockdown of hsa_circRNA_002178 directly decreased the combination of miR-328-3p, and thus downregulated COL1A1 and impaired breast cancer angiogenesis (**Figure 2**). COL1A1 is upregulated in brain metastases (67) and oral squamous cell carcinomas (68), which are potentially associated with angiogenesis. A previous study showed that miR-328-3p directly targets the 3’UTR of matrix metalloprotease 16 (MMP-16) and suppresses its expression in osteosarcoma cells (69). MMP16 is a member of the matrix metalloproteinase (MMP) family (10) and can hydrolyze ECM proteins. MMPs [such as MMP-9 (70)] can break the ECM and cell connections, promoting tumor angiogenesis and progression. HIF-1 mediates the regulation of VEGF and MMPs at the transcriptional level (71). MMPs (such as MMP-2/-3/-7 and -9) promote angiogenesis by degrading the extracellular protein matrix, releasing VEGF without affecting its activity (9, 72, 73). MMP-16 has a similar structure to MMPs, and the 3’UTR of MMP-16 is targeted by miR-328-3P, while hsa_circ_0000519 can adsorb miR-328-3P. Therefore, further investigation is required to see if hsa_circ_0000519 can promote tumor angiogenesis by sponging miR-328-3p to regulate other MMPs.

#### GECs Related circRNAs

**Figure 3:**
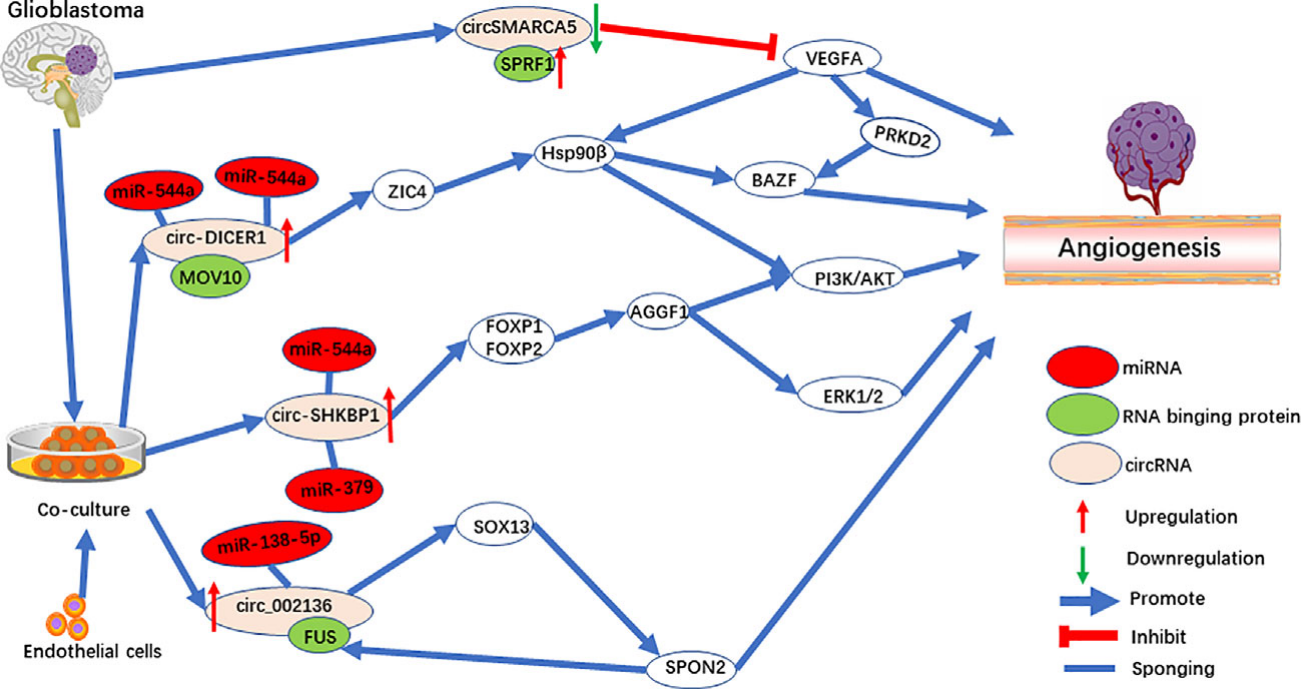
The role of circRNAs in pro-angiogenesis and anti-angiogenesis in glioblastoma.

The method of co-culturing glioblastoma (GBM) cells and endothelial cells was used to explore the cellular communication and molecular adjustment between the two cell types. Three significant upregulated circRNAs [circ-SHKBP1 (48), circ_002136 (49), and circ-DICER1 (50)] were identified (**Figure 3**). Among them, circ-SHKBP1 (circbase ID: hsa_circ_0000936) serves as a sponge for miR-379 and miR-544a, competing with the combination of 3’UTR of FOXP1 and FOXP2. At the same time, FOXP1 and FOXP2 are the angiogenic promoter of AGGF1, which induce tube formation of GECs through the PI3K/AKT and ERK1/2 signaling pathways (**Figure 3**). AGGF1 acts as a angiogenic promoter and has been widely reported in gastric carcinoma (74), hepatocellular carcinoma (75), and medulloblastoma (76). Meanwhile, the activation of the PI3K/AKT and ERK1/2 signaling pathways promoted by AGGF1 has been found when angiogenesis was activated (77–79).

Circ_002136 was found to combine with miR-138-5p, resulting in increased expression of SOX13, which upregulated SPON2 by directly binding the SPON2 promoter region (**Figure 3**). Interestingly, FUS acts as RNA binding protein to upregulate circ_002136, and was upregulated by promotor SPON2, to form a feedback loop. Further research showed that SPON2-knockdown significantly suppresses tumor angiogenesis in GECs. In addition, one of the SPON2 family members inhibited endotheliocyte proliferation, migration, and angiogenesis by inhibiting HIF-1a, VEGFA expression, and the phosphorylation of VEGFR-2 in colon cancer (80). Meanwhile, after IL-1β induced cartilage degradation, overexpressed miR-138-5p was found to be a FOXC1 sponge, and upregulation of MMP-13 was observed (81). Interestingly, SOX13, which was shown to be a target for circ_002136, regulates angiogenesis through a system model of homologous phenotypes (82).

Circ-DICER1 is a target for the RNA binging protein, MOV10, which together regulate angiogenesis (50). circ-DICER1 directly binds the 3’UTR of miR-103a-3p and miR-382-5p and downregulates ZIC4 in GECs (**Figure 3**). Furthermore, ZIC4 promotes tube formation through the PI3K/Akt signaling pathway by upregulating heat shock protein 90β (Hsp90β). In addition, Hsp90β up-regulates the expression of VEGFRs and promotes tumor angiogenesis by VEGFRs promoters in HCC (83, 84). Meanwhile, HSP90β directly targets the BAZF mRNA after activation by the VEGF-A/PRKD2 pathway to promote angiogenesis (85). In another study, miR-103a-3p was also found to target PTEN to promote EPC migration and angiogenesis (86).

#### Exosome has_circRNA_100338

Exosomes have been used as messengers of intercellular communication, in which circRNA plays an essential role in tumorigenesis and progress (87). circRNA-100338 (51) is upregulated in both HCC cells and their secreted exosomes. Based on RNA pulldown assays, circRNA-100338 potentially targets NOVA2 and promotes angiogenesis in HUVECs (**Figure 2**). Furthermore, a previous study has shown that the downregulation of NOVA2 can disrupt angiogenesis (88). In addition, it was found that exosome-derived ncRNAs promote cell communication in the microenvironment and regulate angiogenesis (89–91). Therefore, as a stable ncRNA in exosomes, a novel therapy has a promising future in the mechanism of tumor angiogenesis and prognosis.

### Anti-Angiogenesis Associated circRNA in Tumor

#### CircHIPK3

CircHIPK3 is located on chromosome chr11:33307958-33309057. In a previous study, circHIPK3 serves as oncogene of multiple tumors. However, in bladder cancer (43, 92) and osteosarcoma (93) tissues, circHIPK3 is significantly downregulated, acting as a tumor suppressor gene. Downregulation of circHIPK3 is associated with angiogenesis (43). CircHIPK3 directly targets miR‐558 and suppresses heparinase (HPSE) expression to inhibit angiogenesis in ECs (**Figure 1**). Additionally, studies by Qu et al. (94) and Zheng et al. (95) demonstrated that upregulation of miR-558 in neuroblastoma and gastric cancer cells promoted expression of HPSE by directly activating or weakening the inhibition of HPSE by Smad4. On the one hand, HPSE is highly expressed in cancer tissue but not in mature vascular ECs (96). On the other hand, HPSE significantly cuts off the HS chain in the endothelial cell matrix and stimulates the release of other pro-angiogenic molecules (97). The active bFGF produced by HPSE binds to HS fragments and directly targets endothelial cells to promote angiogenesis (96, 98). Meanwhile, studies have also shown that exogenous HPSE can induce melanoma cells to release VEGF, and this has no correlation with the enzyme activity of HPSE (99).

#### CircSMARCA5

CircSMARCA5 (circbase ID: hsa_circ_0001445) (46, 47) is downregulated in GBM and is negatively correlated with SRSF1 and VEGFA. SPRF1, a splicing factor, has been shown to bind directly to circSMARCA5 to regulate VEGFA expression (**Figure 3**). Furthermore, circSMARCA5 is significantly correlated with vascular microvessel density, suggesting that circSMARCA5 is a potential biomarker for GBM angiogenesis. In addition, circSMARCA5 is downregulated in gastric cancer (100), cervical cancer (101), non-small cell lung cancer (102), hepatocellular carcinoma (103), multiple myeloma (104), and acts as a biomarker. On the contrary, circSMARCA5 is upregulated in prostate cancer (105).

### CircRNA Regulates Angiogenesis in Other Diseases

Upregulation of hsa_circ_0003575 (52) was observed in oxLDL-treated HUVECs to simulate atherosclerosis, and downregulation of hsa_circ_0003575 promoted angiogenesis in HUVECs. In a hypoxia-induced microenvironment, endothelial cells are more prone to angiogenesis, and hsa_circ_0010729 (53) was found to be upregulated and bound miR-186. Knockdown of hsa_circ_0010729 repressed the expression of HIF-1α and inhibited cellular angiogenesis related capacity and promoted apoptosis in HUVECs. Significant upregulation of cZNF609 (54) was observed both *in vivo* and *in vitro* in high glucose-induced microenvironments. Subsequent bio functional experiments demonstrated that cZNF609 inhibited angiogenesis *via* sequestering miR-615-5p and increasing the expression of MEF2A. In an adult mouse model of myocardial infarction, circNfix (55) is regulated by the transcription factor, Meis1-bound superenhancer, thereby promoting angiogenesis and cardiac regeneration. CircHIPK3 (56) is upregulated and served as a sponge for miR-30a-3p, resulting in increased expression of VEGC-C, FZD4 and WNT2, and promoting the formation of new blood vessels in EC. One of the key factors in fracture healing is the recovery of blood flow, and the dysregulation of circRNAs in bone marrow stem cells inhibits angiogenesis. The downregulation of hsa_circ_0074834 (57) releases inhibition of miR-942-5p to upregulate ZEB1 and VEGF, promoting osteogenic differentiation and the repair of bone defects. For patients with preeclampsia, circ_0063517 (58) and ETBR were found downregulated in the placenta tissue; circ_0063517 promotes angiogenesis by sponging miR-31-5p to downregulate ETBR.

### The Potential Therapeutic Role of circRNAs

*In vivo* and *in vitro* experiments have verified that the regulation of the transcriptional patterns of circRNAs is related to the survival and growth of tumor cells. Therefore, there is great potential to use siRNA, ASO, and circRNAs to treat tumors and other diseases. Previous reviews have described a therapeutic role for circRNAs in cardiovascular disease (106). One advantage of circRNAs in its therapeutic role is that it has a stable structure that is not easily degraded compared with other lncRNAs. Additionally, circRNAs have been found in plasma exosomes, which provide a reference model for simulating circRNA as drug targeted delivery *in vivo*. Further, chemical modifications enable gene delivery as a treatment strategy, while minimizing side effects (107).

### CircRNAs Associated Bioinformatics Software

With the development of deep sequencing, a large number of transcripts were discovered, and data were formed and developed into databases for further analysis (**Supplementary Table 1**). These databases record the specific ID, sequences, potential functions, and expression patterns of newly discovered circRNAs in different species in different diseases. Different databases may have different results in predicting the function of circRNAs (such as miRNA binding sites, protein binding sites, and coding proteins) due to differences in algorithms and individual heterogeneity. Therefore, the intersection of results from multiple databases may be a potential method to predict the function of circRNA more accurately.

## Future Prospective and Conclusion

Currently, circRNA plays a significant role in carcinoma angiogenesis through varied biological pathways. Current studies have shown that circRNAs regulate tumor angiogenesis mainly through two pathways. The first is by functioning as a miRNA sponge, thereby upregulating or downregulating downstream genes, and promoting or repressing angiogenesis. Second, RBP directly targets circRNAs to regulate tumor angiogenesis (46, 47, 49, 50). Nevertheless, it has been previously reported that circRNA-encoded proteins regulate GBM malignancy (38). However, current research has not found whether circRNA regulates tumor angiogenesis by encoding proteins. Therefore, there is no doubt that the exploration of circRNAs in tumor angiogenesis is in its infancy, and more detailed analyses is essential.

Tumor angiogenesis is co-regulated by a variety of pro- and anti-angiogenic factors, among which VEGF-related pro-angiogenic factors are the most remarkable (108–110). On the one hand, circRNA-MYLK (42), circHIPK3 (56), and hsa_circ_0074834 (57) act as a miRNA sponge, directly upregulating VEGF expression to promote angiogenesis. On the other hand, circSMARCA5 (46) has been shown to serve as a sponge for SRSF1, negatively regulating VEGF and anti-angiogenesis in GBM. Thus, whether circRNA regulates angiogenesis through angiogenesis-related factors, such as angiopoietin, FGF, MMPs, and Endostatin, requires further exploration.

As a stable RNA, circRNAs have natural advantages and great potential as a diagnostic biomarker (111). Compared with tumor tissue, circRNA in plasma or plasma exosomes have greater clinical significance as biomarkers for tumor diagnosis (112), because a blood test is less likely to lead to metastasis than a biopsy or surgical removal.

Furthermore, monitoring angiogenesis, the necessary process for oncogenesis and carcinoma progression, to diagnose or evaluate prognosis may be a worthwhile direction to explore. The exosome circRNA, has_circRNA_100338 (51), has been shown to promote angiogenesis and to be a potential biomarker for HCC.

However, circRNA displays heterogeneity as a diagnostic biomarker (113), and circRNAs may be inversely expressed in different types of tumors. circHIPK3 serves as oncogene of a variety of tumor such as CC (114–116) and NSLC (117, 119). However, in bladder cancer (43, 92) and osteosarcoma (93) tissues, circHIPK3 is significantly downregulated, repressing invasion and metastasis of tumor cells and predicting a good prognosis. Additionally, circSMARCA5 is downregulated in gastric cancer (100), cervical cancer (101), NSLC (102), HCC (103), multiple myeloma (104), but upregulated in prostate cancer (105). Therefore, whether such heterogeneity also exists in the regulation of angiogenesis by circRNAs needs further exploration.

In summary, we reviewed the biological characteristics, functions, and molecular mechanisms of circRNAs in tumor angiogenesis. We believe that circRNAs have great potential as a target for antiangiogenic therapy and as a diagnostic biomarker in tumors, which deserves our attention in the future.

## Author Contributions

SJ: conception and design. SJ, RF, JS, HW, and JM: wrote and critically reviewed the manuscript. SJ, XH, HC, and JL: figure design and elaboration. NL and ML: directed manuscript. All authors contributed to the article and approved the submitted version.

## Funding

This work was supported by National Natural Science Foundation of China (81400679), Guangdong Natural Science Foundation (2014A030310067), Guangzhou Science and Technology Programs (Grant No. 202002030387; 201704020153), Special fund of Foshan Summit plan[2019D006] and Foundation of Basic and Applied Basic Research of Guangdong Province [2020A1515110073].

## Conflict of Interest

The authors declare that the research was conducted in the absence of any commercial or financial relationships that could be construed as a potential conflict of interest.

## References

[1] Herbert SP Stainier DYR. Molecular control of endothelial cell behaviour during blood vessel morphogenesis. Nat Rev Mol Cell Biol (2011) 12(9):551–64. 10.1038/nrm3176 PMC3319719 21860391

[2] Risau W. Differentiation of endothelium. FASEB J (1995) 9(10):926–33. 7615161

[3] Hanahan D Folkman J. Patterns and emerging mechanisms of the angiogenic switch during tumorigenesis. Cell (1996) 86(3):353–64. 10.1016/s0092-8674(00)80108-7 8756718

[4] V B G C. The angiogenic switch in carcinogenesis. Semin Cancer Biol (2009) 19(5):329–37. 10.1016/j.semcancer.2009.05.003 19482086

[5] Hanahan D Weinberg RA. Hallmarks of cancer: the next generation. Cell (2011) 144(5):646–74. 10.1016/j.cell.2011.02.013 21376230

[6] Senger DR Van de Water L Brown LF Nagy JA Yeo KT Yeo TK. Vascular permeability factor (VPF, VEGF) in tumor biology. Cancer Metastasis Rev (1993) 12(3-4):303–24. 10.1007/bf00665960 8281615

[7] Senger DR Perruzzi CA Feder J Dvorak HF. A highly conserved vascular permeability factor secreted by a variety of human and rodent tumor cell lines. Cancer Res (1986) 46(11):5629–32. 3756910

[8] Fagiani E Christofori G. Angiopoietins in angiogenesis. Cancer Lett (2013) 328(1):18–26. 10.1016/j.canlet.2012.08.018 22922303

[9] Ebrahem Q Chaurasia SS Vasanji A Qi JH Klenotic PA Cutler A. Cross-talk between vascular endothelial growth factor and matrix metalloproteinases in the induction of neovascularization in vivo. Am J Pathol (2010) 176(1):496–503. 10.2353/ajpath.2010.080642 19948826 PMC2797907

[10] Itoh Y. Membrane-type matrix metalloproteinases: Their functions and regulations. Matrix Biol (2015) 44-46:207–23. 10.1016/j.matbio.2015.03.004 25794647

[11] Cross MJ Claesson-Welsh L. FGF and VEGF function in angiogenesis: signalling pathways, biological responses and therapeutic inhibition. Trends Pharmacol Sci (2001) 22(4):201–7. 10.1016/s0165-6147(00)01676-x 11282421

[12] Scholz A Plate KH Reiss Y. Angiopoietin-2: a multifaceted cytokine that functions in both angiogenesis and inflammation. Ann N Y Acad Sci (2015) 1347:45–51. 10.1111/nyas.12726 25773744

[13] Eklund L Kangas J Saharinen P. Angiopoietin-Tie signalling in the cardiovascular and lymphatic systems. Clin Sci (London Engl 1979) (2017) 131(1):87–103. 10.1042/CS20160129 PMC5146956 27941161

[14] Jabłońska-Trypuć A Matejczyk M Rosochacki S. Matrix metalloproteinases (MMPs), the main extracellular matrix (ECM) enzymes in collagen degradation, as a target for anticancer drugs. J Enzyme Inhib Med Ch (2016) 31(sup1):177–83. 10.3109/14756366.2016.1161620 27028474

[15] Fu Y Chen Y Luo X Liang Y Shi H Gao L. The heparin binding motif of endostatin mediates its interaction with receptor nucleolin. Biochemistry (2009) 48(49):11655–63. 10.1021/bi901265z 19877579

[16] Colorado PC Torre A Kamphaus G Maeshima Y Hopfer H Takahashi K. Anti-angiogenic cues from vascular basement membrane collagen. Cancer Res (2000) 60(9):2520–6. 10811134

[17] O’Reilly MS Holmgren L Shing Y Chen C Rosenthal RA Moses M. Angiostatin: a novel angiogenesis inhibitor that mediates the suppression of metastases by a Lewis lung carcinoma. Cell (1994) 79(2):315–28. 10.1016/0092-8674(94)90200-3 7525077

[18] DJ K ML W. Ectopic localization of mitochondrial ATP synthase: a target for anti-angiogenesis intervention? J Bioenerg Biomembr (2005) 37(6):461–5. 10.1007/s10863-005-9492-x 16691484

[19] Sanger HL Klotz G Riesner D Gross HJ Kleinschmidt AK. Viroids are single-stranded covalently closed circular RNA molecules existing as highly base-paired rod-like structures. Proc Natl Acad Sci USA (1976) 73(11):3852–6. 10.1073/pnas.73.11.3852 PMC431239 1069269

[20] Salzman J Gawad C Wang PL Lacayo N Brown PO. Circular RNAs are the predominant transcript isoform from hundreds of human genes in diverse cell types. PloS One (2012) 7(2):e30733. 10.1371/journal.pone.0030733 22319583 PMC3270023

[21] Jeck WR Sorrentino JA Wang K Slevin MK Burd CE Liu J. Circular RNAs are abundant, conserved, and associated with ALU repeats. Rna (2013) 19(2):141–57. 10.1261/rna.035667.112 PMC3543092 23249747

[22] Hansen TB Jensen TI Clausen BH Bramsen JB Finsen B Damgaard CK. Natural RNA circles function as efficient microRNA sponges. Nature (2013) 495(7441):384–8. 10.1038/nature11993 23446346

[23] Memczak S Jens M Elefsinioti A Torti F Krueger J Rybak A. Circular RNAs are a large class of animal RNAs with regulatory potency. Nature (2013) 495(7441):333–8. 10.1038/nature11928 23446348

[24] Guarnerio J Bezzi M Jeong JC Paffenholz SV Berry K Naldini MM. Oncogenic Role of Fusion-circRNAs Derived from Cancer-Associated Chromosomal Translocations. Cell (2016) 165(2):289–302. 10.1016/j.cell.2016.03.020 27040497

[25] Q Z W W Q Z C C W Y J L. Roles of circRNAs in the tumour microenvironment. Mol Cancer (2020) 19(1):14. 10.1186/s12943-019-1125-9 31973726 PMC6977266

[26] Zhang Y Zhang XO Chen T Xiang JF Yin QF Xing YH. Circular intronic long noncoding RNAs. Mol Cell (2013) 51(6):792–806. 10.1016/j.molcel.2013.08.017 24035497

[27] Li Z Huang C Bao C Chen L Lin M Wang X. Exon-intron circular RNAs regulate transcription in the nucleus. Nat Struct Mol Biol (2015) 22(3):256–64. 10.1038/nsmb.2959 25664725

[28] Zhang Y Xue W Li X Zhang J Chen S Zhang JL. The Biogenesis of Nascent Circular RNAs. Cell Rep (2016) 15(3):611–24. 10.1016/j.celrep.2016.03.058 27068474

[29] Gao Y Wang J Zheng Y Zhang J Chen S Zhao F. Comprehensive identification of internal structure and alternative splicing events in circular RNAs. Nat Commun (2016) 7:12060. 10.1038/ncomms12060 27350239 PMC4931246

[30] Zhang XO Dong R Zhang Y Zhang JL Luo Z Zhang J. Diverse alternative back-splicing and alternative splicing landscape of circular RNAs. Genome Res (2016) 26(9):1277–87. 10.1101/gr.202895.115 PMC5052039 27365365

[31] Ashwal-Fluss R Meyer M Pamudurti NR Ivanov A Bartok O Hanan M. circRNA biogenesis competes with pre-mRNA splicing. Mol Cell (2014) 56(1):55–66. 10.1016/j.molcel.2014.08.019 25242144

[32] Taulli R Loretelli C Pandolfi PP. From pseudo-ceRNAs to circ-ceRNAs: a tale of cross-talk and competition. Nat Struct Mol Biol (2013) 20(5):541–3. 10.1038/nsmb.2580 PMC4141855 23649362

[33] Ambros V. The functions of animal microRNAs. Nature (2004) 431(7006):350–5. 10.1038/nature02871 15372042

[34] Turner M Galloway A Vigorito E. Noncoding RNA and its associated proteins as regulatory elements of the immune system. Nat Immunol (2014) 15(6):484–91. 10.1038/ni.2887 24840979

[35] Wang Z Lei X Wu F. Identifying Cancer-Specific circRNA-RBP Binding Sites Based on Deep Learning. Molecules (Basel Switzerland) (2019) 24(22):4035. 10.3390/molecules24224035 PMC6891306 31703384

[36] Zhang M Wang T Xiao G Xie Y. Large-Scale Profiling of RBP-circRNA Interactions from Public CLIP-Seq Datasets. Genes-Basel (2020) 11(1):E54. 10.3390/genes11010054 31947823 PMC7016857

[37] Yang Y Fan X Mao M Song X Wu P Zhang Y. Extensive translation of circular RNAs driven by N(6)-methyladenosine. Cell Res (2017) 27(5):626–41. 10.1038/cr.2017.31 PMC5520850 28281539

[38] Y Y X G M Z S Y C S F X. Novel Role of FBXW7 Circular RNA in Repressing Glioma Tumorigenesis. J Natl Cancer Inst (2018) 110(3). 10.1093/jnci/djx166 PMC6019044 28903484

[39] Pamudurti NR Bartok O Jens M Ashwal-Fluss R Stottmeister C Ruhe L. Translation of CircRNAs. Mol Cell (2017) 66(1):9–21. 10.1016/j.molcel.2017.02.021 28344080 PMC5387669

[40] Annese T Tamma R De Giorgis M Ribatti D. microRNAs Biogenesis, Functions and Role in Tumor Angiogenesis. Front Oncol (2020) 10:581007. 10.3389/fonc.2020.581007 33330058 PMC7729128

[41] Wang Y Wang L Chen C Chu X. New insights into the regulatory role of microRNA in tumor angiogenesis and clinical implications. Mol Cancer (2018) 17(1):22. 10.1186/s12943-018-0766-4 29415727 PMC5804051

[42] Zhong Z Huang M Lv M He Y Duan C Zhang L. Circular RNA MYLK as a competing endogenous RNA promotes bladder cancer progression through modulating VEGFA/VEGFR2 signaling pathway. Cancer Lett (2017) 403:305–17. 10.1016/j.canlet.2017.06.027 28687357

[43] Li Y Zheng F Xiao X Xie F Tao D Huang C. CircHIPK3 sponges miR-558 to suppress heparanase expression in bladder cancer cells. EMBO Rep (2017) 18(9):1646–59. 10.15252/embr.201643581 PMC5579470 28794202

[44] Chen Y Li Z Zhang M Wang B Ye J Zhang Y. Circ-ASH2L promotes tumor progression by sponging miR-34a to regulate Notch1 in pancreatic ductal adenocarcinoma. J Exp Clin Cancer Res (2019) 38(1):466. 10.1186/s13046-019-1436-0 31718694 PMC6852927

[45] Liu T Ye P Ye Y Lu S Han B. Circular RNA hsa_circRNA_002178 silencing retards breast cancer progression via microRNA-328-3p-mediated inhibition of COL1A1. J Cell Mol Med (2020) 24(3):2189–201. 10.1111/jcmm.14875 PMC7011152 31957232

[46] Barbagallo D Caponnetto A Brex D Mirabella F Barbagallo C Lauretta G. CircSMARCA5 Regulates VEGFA mRNA Splicing and Angiogenesis in Glioblastoma Multiforme Through the Binding of SRSF1. Cancers (2019) 11(2):194. 10.3390/cancers11020194 PMC6406760 30736462

[47] Barbagallo D Caponnetto A Cirnigliaro M Brex D Barbagallo C D’Angeli F. CircSMARCA5 Inhibits Migration of Glioblastoma Multiforme Cells by Regulating a Molecular Axis Involving Splicing Factors SRSF1/SRSF3/PTB. Int J Mol Sci (2018) 19(2):480. 10.3390/ijms19020480 PMC5855702 29415469

[48] He Q Zhao L Liu Y Liu X Zheng J Yu H. circ-SHKBP1 Regulates the Angiogenesis of U87 Glioma-Exposed Endothelial Cells through miR-544a/FOXP1 and miR-379/FOXP2 Pathways. Mol Ther Nucleic Acids (2018) 10:331–48. 10.1016/j.omtn.2017.12.014 PMC5862134 29499945

[49] He Z Ruan X Liu X Zheng J Liu Y Liu L. FUS/circ_002136/miR-138-5p/SOX13 feedback loop regulates angiogenesis in Glioma. J Exp Clin Cancer Res (2019) 38(1):65. 10.1186/s13046-019-1065-7 30736838 PMC6368736

[50] He Q Zhao L Liu X Zheng J Liu Y Liu L. MOV10 binding circ-DICER1 regulates the angiogenesis of glioma via miR-103a-3p/miR-382-5p mediated ZIC4 expression change. J Exp Clin Cancer Res (2019) 38(1):9. 10.1186/s13046-018-0990-1 30621721 PMC6323715

[51] Huang X Huang Z Huang J Xu B Huang X Xu Y. Exosomal circRNA-100338 promotes hepatocellular carcinoma metastasis via enhancing invasiveness and angiogenesis. J Exp Clin Cancer Res (2020) 39(1):20. 10.1186/s13046-020-1529-9 31973767 PMC6979009

[52] Li C Ma L Yu B. Circular RNA hsa_circ_0003575 regulates oxLDL induced vascular endothelial cells proliferation and angiogenesis. Biomed Pharmacother (2017) 95:1514–9. 10.1016/j.biopha.2017.09.064 28946214

[53] Dang R Liu F Li Y. Circular RNA hsa_circ_0010729 regulates vascular endothelial cell proliferation and apoptosis by targeting the miR-186/HIF-1α axis. Biochem Bioph Res Co (2017) 490(2):104–10. 10.1016/j.bbrc.2017.05.164 28571741

[54] Liu C Yao M Li C Shan K Yang H Wang J. Silencing Of Circular RNA-ZNF609 Ameliorates Vascular Endothelial Dysfunction. Theranostics (2017) 7(11):2863–77. 10.7150/thno.19353 PMC5562221 28824721

[55] Huang S Li X Zheng H Si X Li B Wei G. Loss of Super-Enhancer-Regulated circRNA Nfix Induces Cardiac Regeneration After Myocardial Infarction in Adult Mice. Circulation (2019) 139(25):2857–76. 10.1161/CIRCULATIONAHA.118.038361 PMC6629176 30947518

[56] Shan K Liu C Liu B Chen X Dong R Liu X. Circular Noncoding RNA HIPK3 Mediates Retinal Vascular Dysfunction in Diabetes Mellitus. Circulation (2017) 136(17):1629–42. 10.1161/CIRCULATIONAHA.117.029004 28860123

[57] Ouyang Z Tan T Zhang X Wan J Zhou Y Jiang G. CircRNA hsa_circ_0074834 promotes the osteogenesis-angiogenesis coupling process in bone mesenchymal stem cells (BMSCs) by acting as a ceRNA for miR-942-5p. Cell Death Dis (2019) 10(12):932. 10.1038/s41419-019-2161-5 31804461 PMC6895238

[58] Li W Yu N Fan L Chen S Wu J. Circ_0063517 acts as ceRNA, targeting the miR-31-5p-ETBR axis to regulate angiogenesis of vascular endothelial cells in preeclampsia. Life Sci (2020) 244:117306. 10.1016/j.lfs.2020.117306 31953159

[59] H Z M B D T L R W X Q Y. Cell-derived microvesicles mediate the delivery of miR-29a/c to suppress angiogenesis in gastric carcinoma. Cancer Lett (2016) 375(2):331–9. 10.1016/j.canlet.2016.03.026 27000664

[60] Jia P Cai H Liu X Chen J Ma J Wang P. Long non-coding RNA H19 regulates glioma angiogenesis and the biological behavior of glioma-associated endothelial cells by inhibiting microRNA-29a. Cancer Lett (2016) 381(2):359–69. 10.1016/j.canlet.2016.08.009 27543358

[61] Wang J Wang Y Wang Y Ma Y Lan Y Yang X. Transforming growth factor β-regulated microRNA-29a promotes angiogenesis through targeting the phosphatase and tensin homolog in endothelium. J Biol Chem (2013) 288(15):10418–26. 10.1074/jbc.M112.444463 PMC3624424 23426367

[62] Dai Y Li D Chen X Tan X Gu J Chen M. Circular RNA Myosin Light Chain Kinase (MYLK) Promotes Prostate Cancer Progression through Modulating Mir-29a Expression. Med Sci Monit (2018) 24:3462–71. 10.12659/MSM.908009 PMC5996838 29798970

[63] Li Z Hu Y Zeng Q Wang H Yan J Li H. Circular RNA MYLK promotes hepatocellular carcinoma progression by increasing Rab23 expression by sponging miR-362-3p. Cancer Cell Int (2019) 19:211. 10.1186/s12935-019-0926-7 31413665 PMC6688277

[64] Li J Gong J Li X Shen L Xie Y Zhang R. MicroRNA-34a promotes CMECs apoptosis and upregulate inflammatory cytokines, thus worsening CMECs damage and inhibiting angiogenesis by negatively targeting the Notch signaling pathway. J Cell Biochem (2018) 10–1002. 10.1002/jcb.27433 30335902

[65] Shi S Jin Y Song H Chen X. MicroRNA-34a attenuates VEGF-mediated retinal angiogenesis via targeting Notch1. Biochem Cell Biol (2019) 97(4):423–30. 10.1139/bcb-2018-0304 30571142

[66] Kumar B Yadav A Lang J Teknos TN Kumar P. Dysregulation of microRNA-34a expression in head and neck squamous cell carcinoma promotes tumor growth and tumor angiogenesis. PloS One (2012) 7(5):e37601. 10.1371/journal.pone.0037601 22629428 PMC3358265

[67] Ilhan-Mutlu A Siehs C Berghoff AS Ricken G Widhalm G Wagner L. Expression profiling of angiogenesis-related genes in brain metastases of lung cancer and melanoma. Tumour Biol (2016) 37(1):1173–82. 10.1007/s13277-015-3790-7 26277786

[68] Sobrinho Santos EM Guimarães TA Santos HO Cangussu LMB de Jesus SF Fraga CADC. Leptin acts on neoplastic behavior and expression levels of genes related to hypoxia, angiogenesis, and invasiveness in oral squamous cell carcinoma. Tumour Biol (2017) 39(5):1393390534. 10.1177/1010428317699130 28459203

[69] Zhang M Zhang J Zhou Q. Elevated expression of microRNA-328-3p suppresses aggressive malignant behaviors via targeting matrix metalloprotease 16 in osteosarcoma. Onco Targets Ther (2019) 12:2063–70. 10.2147/OTT.S195022 PMC6430066 30936722

[70] Zhang G Miyake M Lawton A Goodison S Rosser CJ. Matrix metalloproteinase-10 promotes tumor progression through regulation of angiogenic and apoptotic pathways in cervical tumors. BMC Cancer (2014) 14:310. 10.1186/1471-2407-14-310 24885595 PMC4022983

[71] Deryugina EI Quigley JP. Tumor angiogenesis: MMP-mediated induction of intravasation- and metastasis-sustaining neovasculature. Matrix Biol (2015) 44-46:94–112. 10.1016/j.matbio.2015.04.004 25912949 PMC5079283

[72] Hawinkels LJAC Zuidwijk K Verspaget HW de Jonge-Muller ESM van Duijn W Ferreira V. VEGF release by MMP-9 mediated heparan sulphate cleavage induces colorectal cancer angiogenesis. Eur J Cancer (Oxford Engl 1990) (2008) 44(13):1904–13. 10.1016/j.ejca.2008.06.031 18691882

[73] Hashimoto G Inoki I Fujii Y Aoki T Ikeda E Okada Y. Matrix metalloproteinases cleave connective tissue growth factor and reactivate angiogenic activity of vascular endothelial growth factor 165. J Biol Chem (2002) 277(39):36288–95. 10.1074/jbc.M201674200 12114504

[74] Yao H Wang B Wu Y Huang Q. High Expression of Angiogenic Factor with G-Patch and FHA Domain1 (AGGF1) Predicts Poor Prognosis in Gastric Cancer. Med Sci Monit (2017) 23:1286–94. 10.12659/msm.903248 PMC5362190 28289272

[75] Wang W Li G Zhu J Huang D Zhou H Zhong W. Overexpression of AGGF1 is correlated with angiogenesis and poor prognosis of hepatocellular carcinoma. Med Oncol (Northwood London England) (2015) 32(4):131. 10.1007/s12032-015-0574-2 25796501

[76] Thompson EM Keir ST Venkatraman T Lascola C Yeom KW Nixon AB. The role of angiogenesis in Group 3 medulloblastoma pathogenesis and survival. Neuro-Oncology (2017) 19(9):1217–27. 10.1093/neuonc/nox033 PMC5570262 28379574

[77] Zhang T Yao Y Wang J Li Y He P Pasupuleti V. Haploinsufficiency of Klippel-Trenaunay syndrome gene Aggf1 inhibits developmental and pathological angiogenesis by inactivating PI3K and AKT and disrupts vascular integrity by activating VE-cadherin. Hum Mol Genet (2016) 25(23):5094–110. 10.1093/hmg/ddw273 PMC6078640 27522498

[78] Chen D Li L Tu X Yin Z Wang Q. Functional characterization of Klippel-Trenaunay syndrome gene AGGF1 identifies a novel angiogenic signaling pathway for specification of vein differentiation and angiogenesis during embryogenesis. Hum Mol Genet (2013) 22(5):963–76. 10.1093/hmg/dds501 23197652

[79] Liu Y Yang H Song L Li N Han Q Tian C. AGGF1 protects from myocardial ischemia/reperfusion injury by regulating myocardial apoptosis and angiogenesis. Apoptosis (2014) 19(8):1254–68. 10.1007/s10495-014-1001-4 24893993

[80] Wang L Liu Y Yang B Li P Cheng X Xiao C. The extracellular matrix protein mindin attenuates colon cancer progression by blocking angiogenesis via Egr-1-mediated regulation. Oncogene (2018) 37(5):601–15. 10.1038/onc.2017.359 28991232

[81] Yuan Y Zhang GQ Chai W Ni M Xu C Chen JY. Silencing of microRNA-138-5p promotes IL-1β-induced cartilage degradation in human chondrocytes by targeting FOXC1: miR-138 promotes cartilage degradation. Bone Joint Res (2016) 5(10):523–30. 10.1302/2046-3758.510.BJR-2016-0074.R2 PMC5108353 27799147

[82] McGary KL Park TJ Woods JO Cha HJ Wallingford JB Marcotte EM. Systematic discovery of nonobvious human disease models through orthologous phenotypes. Proc Natl Acad Sci USA (2010) 107(14):6544–9. 10.1073/pnas.0910200107 PMC2851946 20308572

[83] Meng J Liu Y Han J Tan Q Chen S Qiao K. Hsp90β promoted endothelial cell-dependent tumor angiogenesis in hepatocellular carcinoma. Mol Cancer (2017) 16(1):72. 10.1186/s12943-017-0640-9 28359326 PMC5374580

[84] Meng J Chen S Lei Y Han J Zhong W Wang X. Hsp90β promotes aggressive vasculogenic mimicry via epithelial-mesenchymal transition in hepatocellular carcinoma. Oncogene (2019) 38(2):228–43. 10.1038/s41388-018-0428-4 30087438

[85] Miwa D Sakaue T Inoue H Takemori N Kurokawa M Fukuda S. Protein kinase D2 and heat shock protein 90 beta are required for BCL6-associated zinc finger protein mRNA stabilization induced by vascular endothelial growth factor-A. Angiogenesis (2013) 16(3):675–88. 10.1007/s10456-013-9345-x 23515950

[86] Zhang P Zhao Q Gong K Long Y Zhang J Li Y. Downregulation of miR-103a-3p Contributes to Endothelial Progenitor Cell Dysfunction in Deep Vein Thrombosis Through PTEN Targeting. Ann Vasc Surg (2019), S890–5096. 10.1016/j.avsg.2019.10.048 31639479

[87] Fanale D Taverna S Russo A Bazan V. Circular RNA in Exosomes. Adv Exp Med Biol (2018) 1087:109–17. 10.1007/978-981-13-1426-1_9 30259361

[88] Giampietro C Deflorian G Gallo S Di Matteo A Pradella D Bonomi S. The alternative splicing factor Nova2 regulates vascular development and lumen formation. Nat Commun (2015) 6:8479. 10.1038/ncomms9479 26446569 PMC4633719

[89] Zhang J Li S Li L Li M Guo C Yao J. Exosome and exosomal microRNA: trafficking, sorting, and function. Genomics Proteomics Bioinformatics (2015) 13(1):17–24. 10.1016/j.gpb.2015.02.001 25724326 PMC4411500

[90] Qin Y Sun R Wu C Wang L Zhang C. Exosome: A Novel Approach to Stimulate Bone Regeneration through Regulation of Osteogenesis and Angiogenesis. Int J Mol Sci (2016) 17(5):712. 10.3390/ijms17050712 PMC4881534 27213355

[91] Mashouri L Yousefi H Aref AR Ahadi AM Molaei F Alahari SK. Exosomes: composition, biogenesis, and mechanisms in cancer metastasis and drug resistance. Mol Cancer (2019) 18(1):75. 10.1186/s12943-019-0991-5 30940145 PMC6444571

[92] F X Y L M W C H D T F Z. Circular RNA BCRC-3 suppresses bladder cancer proliferation through miR-182-5p/p27 axis. Mol Cancer (2018) 17(1):144. 10.1186/s12943-018-0892-z 30285878 PMC6169039

[93] M X Z K Z C. Circular RNA circ_HIPK3 is down-regulated and suppresses cell proliferation, migration and invasion in osteosarcoma. J Cancer (2018) 9(10):1856–62. 10.7150/jca.24619 PMC5968774 29805712

[94] Qu H Zheng L Pu J Mei H Xiang X Zhao X. miRNA-558 promotes tumorigenesis and aggressiveness of neuroblastoma cells through activating the transcription of heparanase. Hum Mol Genet (2015) 24(9):2539–51. 10.1093/hmg/ddv018 25616966

[95] Zheng L Jiao W Song H Qu H Li D Mei H. miRNA-558 promotes gastric cancer progression through attenuating Smad4-mediated repression of heparanase expression. Cell Death Dis (2016) 7(9):e2382. 10.1038/cddis.2016.293 27685626 PMC5059886

[96] Elkin M Ilan N Ishai-Michaeli R Friedmann Y Papo O Pecker I. Heparanase as mediator of angiogenesis: mode of action. FASEB J (2001) 15(9):1661–3. 10.1096/fj.00-0895fje 11427519

[97] Vlodavsky I Miao HQ Medalion B Danagher P Ron D. Involvement of heparan sulfate and related molecules in sequestration and growth promoting activity of fibroblast growth factor. Cancer metastasis Rev (1996) 15(2):177–86. 10.1007/bf00437470 8842489

[98] Masola V Zaza G Gambaro G Franchi M Onisto M. Role of heparanase in tumor progression: Molecular aspects and therapeutic options. Semin Cancer Biol (2019) S1044–579. 10.1016/j.semcancer.2019.07.014 31348993

[99] Hoß SG Grundmann M Benkel T Gockel L Schwarz S Kostenis E. Pro-Angiogenic Effects of Latent Heparanase and Thrombin Receptor-Mediated Pathways-Do They Share a Common Ground in Melanoma Cells? Thromb Haemost (2018) 118(10):1803–14. 10.1055/s-0038-1669922 30235481

[100] Cai J Chen Z Zuo X. circSMARCA5 Functions as a Diagnostic and Prognostic Biomarker for Gastric Cancer. Dis Markers (2019) 2019:2473652. 10.1155/2019/2473652 30956729 PMC6431400

[101] Tian JDC Liang L. Involvement of circular RNA SMARCA5/microRNA-620 axis in the regulation of cervical cancer cell proliferation, invasion and migration. Eur Rev Med Pharmacol (2018) 22(24):8589–98. 10.26355/eurrev_201812_16622 30575898

[102] Wang Y Li H Lu H Qin Y. Circular RNA SMARCA5 inhibits the proliferation, migration, and invasion of non-small cell lung cancer by miR-19b-3p/HOXA9 axis. Onco Targets Ther (2019) 12:7055–65. 10.2147/OTT.S216320 PMC6722457 31564891

[103] Li Z Zhou Y Yang G He S Qiu X Zhang L. Using circular RNA SMARCA5 as a potential novel biomarker for hepatocellular carcinoma. Clin Chim Acta (2019) 492:37–44. 10.1016/j.cca.2019.02.001 30716279

[104] Liu H Wu Y Wang S Jiang J Zhang C Jiang Y. Circ-SMARCA5 suppresses progression of multiple myeloma by targeting miR-767-5p. BMC Cancer (2019) 19(1):937. 10.1186/s12885-019-6088-0 31601173 PMC6785934

[105] Kong Z Wan X Zhang Y Zhang P Zhang Y Zhang X. Androgen-responsive circular RNA circSMARCA5 is up-regulated and promotes cell proliferation in prostate cancer. Biochem Bioph Res Co (2017) 493(3):1217–23. 10.1016/j.bbrc.2017.07.162 28765045

[106] Holdt LM Kohlmaier A Teupser D. Circular RNAs as Therapeutic Agents and Targets. Front Physiol (2018) 9:1262. 10.3389/fphys.2018.01262 30356745 PMC6189416

[107] Patil S Gao YG Lin X Li Y Dang K Tian Y. The Development of Functional Non-Viral Vectors for Gene Delivery. Int J Mol Sci (2019) 20(21). 10.3390/ijms20215491 PMC6862238 31690044

[108] Claesson‐Welsh L Welsh M. VEGFA and tumour angiogenesis. J Intern Med (2013) 273(2):114–27. 10.1111/joim.12019 23216836

[109] Shibuya M. VEGF-VEGFR System as a Target for Suppressing Inflammation and other Diseases. Endocr Metab Immune Disord Drug Targets (2015) 15(2):135–44. 10.2174/1871530315666150316121956 25772179

[110] Ranieri G Patruno R Ruggieri E Montemurro S Valerio P Ribatti D. Vascular endothelial growth factor (VEGF) as a target of bevacizumab in cancer: from the biology to the clinic. Curr Med Chem (2006) 13(16):1845–57. 10.2174/092986706777585059 16842197

[111] Chen B Huang S. Circular RNA: An emerging non-coding RNA as a regulator and biomarker in cancer. Cancer Lett (2018) 418:41–50. 10.1016/j.canlet.2018.01.011 29330104

[112] Bai H Lei K Huang F Jiang Z Zhou X. Exo-circRNAs: a new paradigm for anticancer therapy. Mol Cancer (2019) 18(1):56. 10.1186/s12943-019-0986-2 30925885 PMC6441195

[113] Li J Li H Lv X Yang Z Gao M Bi Y. Diagnostic performance of circular RNAs in human cancers: A systematic review and meta-analysis. Mol Genet Genomic Med (2019) 7(7):e749. 10.1002/mgg3.749 PMC6625099 31106993

[114] Yan Y Meng S Baoli Q. CircHIPK3 promotes colorectal cancer cells proliferation and metastasis via modulating of miR-1207-5p/FMNL2 signal. Biochem Biophys Res Commun (2020) 524(4):839–46. 10.1016/j.bbrc.2020.01.055 32046858

[115] Zeng K Chen X Xu M Liu X Hu X Xu T. CircHIPK3 promotes colorectal cancer growth and metastasis by sponging miR-7. Cell Death Dis (2018) 9(4):417. 10.1038/s41419-018-0454-8 29549306 PMC5856798

[116] Yanli Z Chen L Xinfeng L Yanlei W Rui Z Yongmei Y. circHIPK3 promotes oxaliplatin-resistance in colorectal cancer through autophagy by sponging miR-637. EBioMedicine (2019) 48:277–88. 10.1016/j.ebiom.2019.09.051 PMC6838436 31631038

[117] Yu H Chen Y Jiang P. Circular RNA HIPK3 exerts oncogenic properties through suppression of miR-124 in lung cancer. Biochem Biophys Res Commun (Biochem. Biophys Res Commun.) (2018) 506(3):455–62. 10.1016/j.bbrc.2018.10.087 30352682

[118] Lu H Han X Ren J Ren K Li Z Sun Z. Circular RNA HIPK3 induces cell proliferation and inhibits apoptosis in non-small cell lung cancer through sponging miR-149. Cancer Biol Ther (2020) 21(2):113–21. 10.1080/15384047.2019.1669995 PMC7012091 31597523

