# Supplementary material for: CircRNA-Mediated Regulation of Angiogenesis: A New Chapter in Cancer Biology
Source: Front Oncol. 2021 Mar 10;11:553706. doi: 10.3389/fonc.2021.553706 (PMC7988083; doi:10.3389/fonc.2021.553706)
Supplement: Supplementary file 1 [file DataSheet_1.pdf]

Supplementary Table 1. Software commonly used to explore circRNAs.

Num	Name	Website	Function	Ref
1	circBase	http://www.circbase.org/	The circRNAs from human, mouse and other species were collected, and the find_CIRc software was used to predict the circRNAs from the ribosomal library.	[1]
2	CircNet	http://circnet.mbc.nctu.edu.tw/	A database based on systematic analysis of transcriptome data from 464 samples	[2]
3	CircInteractome	https://circinteractome.nia.nih.gov/	This database predicts the binding sites of known RNA-binding protein datasets and circRNA in circBase.	[3]
4	CIRCpedia	http://www.picb.ac.cn/rnomics/circpedia/	The database provided information such as the origin gene of circRNA ID, corresponding linear transcript, expression quantity, starting and ending location of exons, cell line, and conservatism.	[4]
5	cRNADb	http://reprod.njmu.edu.cn/cgi-bin/circrnadb/circRNADb.php	A database summarizing the protein-coding human circRNAs.	NA
6	deepBase	http://rna.sysu.edu.cn/deepbase3/	This database annotates and identifies circRNA/sRNA et al and their expression patterns from the new generation sequencing technology data	[5]
7	circRNA Disease	http://cgga.org.cn:9091/circRNADisease/	The database was constructed mainly through literature retrieval of all relevant research results of circRNA and disease	[6]
8	MiOncoCirc	https://mioncocirc.github.io/	The database links circrNA to clinical symptoms and disease.	[7]
9	cir2traits	http://gyanxet-beta.com/circdb/	The database collects circRNA related to human diseases	[8]
10	CSCD	http://gb.whu.edu.cn/CSCD/	CSCD contains tumor-specific circrnas	[9]
11	CircFunBase	http://bis.zju.edu.cn/CircFunBaseBlast/	Based on qRT-PCR, the expression patterns of circleRNA in dozens of diseases were provided	NA
12	BIOINF	http://www.bioinf.com.cn/	Primer design for circRNA	[10]
13	exoRBase	www.exoRBase.org	Lncrnas in plasma exosomes were included in this database	[11]

Reference

[1] Glažar P, Papavasileiou P, Rajewsky N (2014). circBase: a database for circular RNAs. Rna, 20:1666-1670.

[2] Liu YC, Li JR, Sun CH, Andrews E, Chao RF, Lin FM, *et al.* (2016). CircNet: a database of circular RNAs derived from transcriptome sequencing data. Nucleic Acids Res, 44:D209-D215.

[3] Dudekula DB, Panda AC, Grammatikakis I, De S, Abdelmohsen K, Gorospe M (2016). CircInteractome: A web tool for exploring circular RNAs and their interacting proteins and microRNAs. RNA Biol, 13:34-42.

[4] Dong R, Ma XK, Li GW, Yang L (2018). CIRCpedia v2: An Updated Database for Comprehensive Circular RNA Annotation and Expression Comparison. Genomics Proteomics Bioinformatics, 16:226-233.

[5] Zheng LL, Li JH, Wu J, Sun WJ, Liu S, Wang ZL, *et al.* (2016). deepBase v2.0: identification, expression, evolution and function of small RNAs, LncRNAs and circular RNAs from deep-sequencing data. Nucleic Acids Res, 44:D196-D202.

[6] Zhao Z, Wang K, Wu F, Wang W, Zhang K, Hu H, *et al.* (2018). circRNA disease: a manually curated database of experimentally supported circRNA-disease associations. Cell Death Dis, 9:475.

[7] Robinson DR, Wu YM, Lonigro RJ, Vats P, Cobain E, Everett J, *et al.* (2017). Integrative clinical genomics of metastatic cancer. Nature, 548:297-303.

[8] Ghosal S, Das S, Sen R, Basak P, Chakrabarti J (2013). Circ2Traits: a comprehensive database for circular RNA potentially associated with disease and traits. Front Genet, 4:283.

[9] Xia S, Feng J, Chen K, Ma Y, Gong J, Cai F, *et al.* (2018). CSCD: a database for cancer-specific circular RNAs. Nucleic Acids Res, 46:D925-D929.

[10] Zhong S, Wang J, Zhang Q, Xu H, Feng J (2018). CircPrimer: a software for annotating circRNAs and determining the specificity of circRNA primers. BMC Bioinformatics, 19:292.

[11] Li S, Li Y, Chen B, Zhao J, Yu S, Tang Y, *et al.* (2018). exoRBase: a database of circRNA, lncRNA and mRNA in human blood exosomes. Nucleic Acids Res, 46:D106-D112.
